# The effect of glutamine on Dehydroepiandrosterone-induced polycystic ovary syndrome rats

**DOI:** 10.1186/s13048-020-00650-7

**Published:** 2020-05-09

**Authors:** Gengxiang Wu, Xue Hu, Jinli Ding, Jing Yang

**Affiliations:** 1 Reproductive Medical Centre, Renmin Hospital of Wuhan University, Wuhan, 430060 Hubei Province People’s Republic of China; 2 Hubei Clinical Research Center for Assisted Reproductive Technology and Embryonic Development, Wuhan, 430060 People’s Republic of China; 3 Reproductive Medical Centre, Taihe Hospital, Shiyan, 442000 Hubei Province People’s Republic of China

**Keywords:** Polycystic ovary syndrome, Dehydroepiandrosterone, Glutamine, Inflammatory factor, Oxidative stress

## Abstract

**Background:**

Previous studies have shown that chronic inflammation and oxidative stress may play an important role in the pathophysiology of polycystic ovary syndrome (PCOS), and glutamine (Gln) have showed the anti-inflammatory and antioxidant properties. So the aim of this study is to investigate the effect of glutamine supplementation on PCOS rats.

**Methods:**

Female Sprague–Dawley rats were randomly assigned into four groups (*n* = 10 /group), control group, PCOS group, PCOS+ 0.5 g/kg Gln group and PCOS+ 1.0 g/kg Gln group. All the PCOS rats were administrated with 6 mg/100 g dehydroepiandrosterone (DHEA) for 20 consecutive days, all the PCOS+Gln groups were intraperitoneal injected glutamine twice in the next morning after the last DHEA injection. All the samples were collected 12 h after the last administration. Ovarian histological examinations were analyzed and the concentration of serum hormone, inflammatory and oxidative stress factors were measured.

**Results:**

There was no obvious ovarian histological change among the PCOS group and PCOS+Gln groups. All the detected inflammation factors [C-reactive protein, interleukin (IL)-6, IL-18, tumor necrosis factor] showed significantly higher in all the PCOS groups compared to the control group (*P* < 0.01), and were significantly decreased with the supplementation of 0.5 g/kg glutamine (*P* < 0.01). Concentrations of superoxide dismutase were significantly lower in all the PCOS groups (*P* < 0.01) compared to the control group, and increased significantly with the supplementation of 0.5 g/kg glutamine (*P* < 0.01). Serum concentrations of malondialdehyde, nitric oxide synthase and nitric oxide were significantly higher in PCOS group (*P* < 0.01) compared with the control group, and significantly decreased to the comparative levels of control group with supplementation of 0.5 g/kg glutamine (*P* < 0.01).

**Conclusion:**

There is low-grade inflammation and oxidative stress in DHEA-induced PCOS rats. The supplementation of 0.5 g/kg glutamine could effectively ameliorate the inflammation and oxidative stress conditions of PCOS.

## Introduction

Polycystic ovary syndrome (PCOS) is one of the most common endocrine diseases in women of reproductive age, with a prevalence ranging from 9 to 18% [1]. This syndrome is characterized by ovulatory dysfunction, polycystic ovarian morphology, insulin resistance and hyperandrogenism [2]. However, the pathogenesis is still unknown.

Previous studies have shown that chronic inflammation and oxidative stress may play an important role on the pathophysiology of PCOS [2–4]. Chronic inflammation is characterized by an increasing in proinflammatory cytokines and chemokines which is associated with insulin resistance [5]. There are researches have revealed that serum oxidative markers are significantly increased in patients with PCOS compared with the normal [6]. Therefore, supplementation with compounds containing anti-inflammatory and antioxidant properties may be effective in improving the pathological condition in PCOS.

Recently, extensive scientific attention has been paid on glutamine (Gln), which is the most abundant amino acid in the body, including plasma and skeletal muscles, and has anti-inflammatory and antioxidant effects as well as modulating the heat-shock protein response during stress [7, 8]. Moreover, glutamine levels will be decreased under stress, which will activate inflammatory processes, raising the rate of protein degradation [8].

Considering the anti-inflammatory and antioxidant properties of glutamine, the present study aimed at investigating the effect of glutamine supplementation on PCOS rats.

## Materials and methods

### Animals and treatments

Forty female Sprague–Dawley (SD) rats (aged 23 days) weighing 80-90 g were obtained from the Laboratory Animal Centre of Wuhan University (Wuhan, China). The rats were maintained in groups in a well-ventilated room at 22 ± 2 °C, on a 12 h light/12 h dark cycle with free access to food and water. The rats were randomly assigned into four groups (*n* = 10 /group), control group, PCOS group, PCOS+ 0.5 g/kg Gln group and PCOS+ 1.0 g/kg Gln group. The PCOS rat model was established according to our previous study [9]. Briefly, all the PCOS rats were daily injected (subcutaneously) with 6 mg/100 g dehydroepiandrosterone (DHEA) (Aladdin Reagent Co., Ltd., Shanghai, China) which was dissolved in olive oil for 20 consecutive days, the control group was injected with an equivalent olive oil for 20 consecutive days. Glutamine (Double-Crane Pharmaceutical Co., Ltd., Beijing, China) was administered by intraperitoneal injection in the next morning after the last DHEA injection, the second injection of glutamine was administered 4 h later, PCOS group and control group were given the same amount of saline. The investigation was conducted in accordance with NIH guidelines (NIH Pub. No.85–23, revised 1996) and was approved by Animal Care and Use Committee of the Wuhan University.

### Sample prep2aration

Twelve hours after the last administration, all the rats were anesthetized with isoflurane on the morning fasting state. The ovarian were fixed in 4% paraformaldehyde in 0.1 M phosphate buffer (pH 7.4) for 6-8 h, dehydrated in the ascending series of ethanol, clarified in xylene and embedded in paraffin. Blood was collected through cardiac puncture into non-heparinized sample tube and centrifuged at 2318×g for 20 min at room temperature and the serum was stored in − 80 °C until required for biochemical assay. All the rats were euthanized after all the samples were collected.

### Histology

The ovaries embedded in paraffin were sliced into 4 μm-thick sections. The sections were deparaffinized with xylene and rehydrated with descending series of ethanol, and stained by hematoxylin and eosin (H&E) according to standard procedures (G1005, Servicebio, Wuhan, China).

### Biochemical analysis

Enzyme-linked immunosorbent assay (ELISA) kits were used to measure the serum concentrations of hormones [follicle stimulating hormone (FSH), luteinizing hormone (LH), testosterone (T), estradiol (E2), progesterone (P), insulin], inflammatory factors [C-reactive protein (CRP), interleukin (IL)-6, IL-18, tumor necrosis factor (TNF)-α], and oxidative stress factors [superoxide dismutase (SOD), malondialdehyde (MDA), nitric oxide synthase (NOS) and nitric oxide (NO)]. The assay kits obtained from Elabscience® (Wuhan, China) for T (E-EL-0072c), CRP (E-EL-R0022c), IL-6(E-EL-R0015c), IL-18(E-EL-R0567c), TNF-α (E-EL-R0019c), Cusabio Biotech CO.,Ltd. (Wuhan, China) for FSH (CSB-E06869r), LH (CSB-E12654r), E2 (CSB-E05110r), P (CSB-E07282r), insulin (CSB-E05070r), Nanjing Jiancheng Bioengineering Institute (Nanjing, China) for SOD(A001–1), MDA(A003–1), NOS(A014–2), NO(A013–2). There were standard curves used to calculate for all these kits. The coefficients of variation within and between plates were less than 10%. The fasting blood glucose (FBG) level was detected by blood glucose meter (SAFE-ACCU, Sinocare, Shenzhen, China) according to the manual. Homeostasis model assessment of insulin resistance (HOMA-IR) was calculated by (concentration of serum insulin × FBG)/22.5.

### Statistical analysis

Data was analyzed using SPSS version 16.0. Mean value and SD were calculated with conventional method. Comparisons among groups were carried out by one-way ANOVA, multiple comparisons between groups were carried out by LSD test. A *P* value below 0.05 was considered to be statistically significant.

## Results

### Pathological observations

**Fig. 1:**
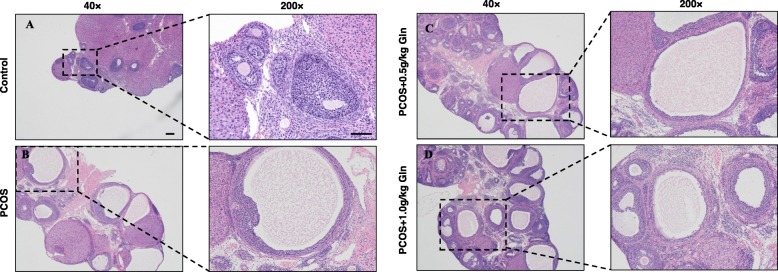
Pathological images of ovarian tissues in control (**a**), PCOS (**b**)and PCOS+ 0.5 kg/kg Gln group (**c**) and PCOS+ 1.0 kg/kg Gln group (**d**). Hematoxylin and eosin staining, (magnification: left pictures × 40, right pictures × 200)

The ovarian weight in the PCOS groups [PCOS group:(12.1 ± 1.2) mg, PCOS+ 0.5 g/kg Gln group: (12.6 ± 1.5) mg, PCOS+ 1.0 g/kg Gln group:(12.4 ± 1.8) mg] showed significantly higher than the controls [(6.6 ± 0.5) mg] (*P*<0.05), however, there was no significance among all the PCOS groups. The ovaries in control group showed normal ovarian morphology and there were follicles and luteal formation at different stages of development, the latter is an indicator of ovulation. The ovaries in PCOS group and PCOS+Gln groups showed a large number of cystic dilatation and fewer corpus luteums, however, there was no obvious change among the PCOS group and PCOS+Gln groups (Fig. 1).

### Serum hormones

**Fig. 2:**
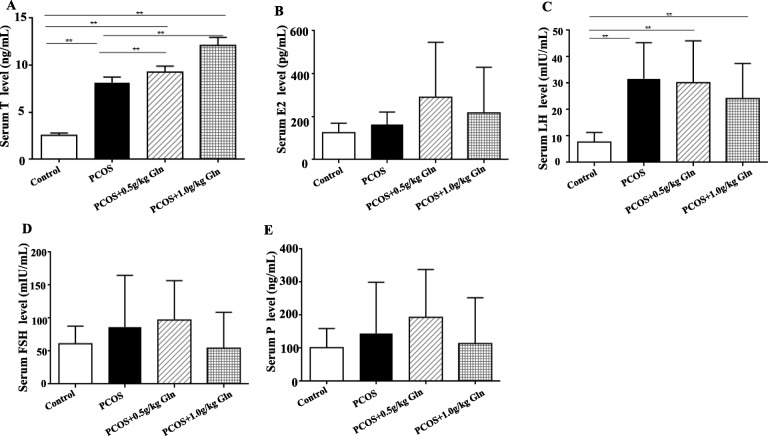
The serum concentrations of hormones. **a**: Serum T levels; **b**: Serum E2 levels; **c**: Serum LH levels; **d**: Serum FSH levels; **e**: Serum P levels. *n* = 10, **P < 0.01

Serum concentrations of T and LH were significantly higher in all the PCOS groups (include PCOS group and PCOS+Gln groups) than the control group (Fig. 2a, c), and the concentrations of T were increasing with the dosage of supplementation of glutamine, but the concentrations of LH were decreasing with the dosage of supplementation of glutamine. However, the concentrations of E2, FSH and P didn’t show significant difference among all the groups (Fig. 2b, d, f).

### Serum insulin and fasting blood glucose

**Fig. 3:**
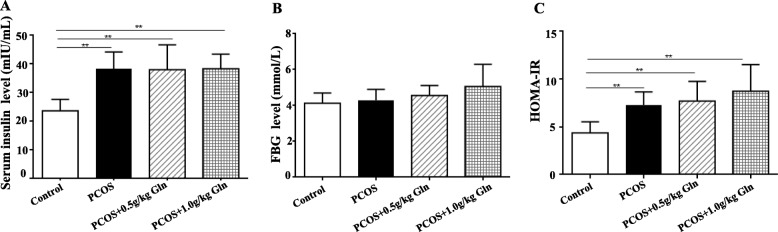
The serum concentrations of insulin and fasting blood glucose (FBG). **a**: insulin; **b**: fasting blood glucose; **c**: HOMA-IR. *n* = 10, **P < 0.01

Serum concentrations of insulin were significantly higher in all the PCOS groups than the control group (*P* < 0.01, Fig. 3a), and the HOMA-IR showed the same tendency (Fig. 3c), although the fasting blood glucose (FBG) didn’t show significant difference among the control and all PCOS groups (*P* > 0.05, Fig. 3b).

### Serum inflammation factors

**Fig. 4:**
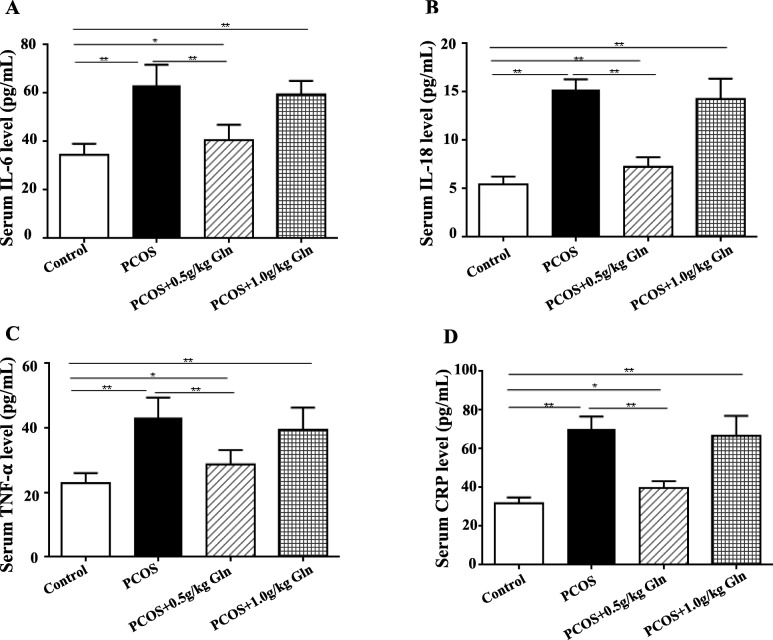
The serum concentrations of inflammatory factors. **a**: Serum IL-6 levels; **b**: Serum IL-18 levels; **c**: Serum TNF- α levels; **d**: Serum CRP levels. *n* = 10, *P < 0.05, **P < 0.001

All serum concentrations of the inflammation factors (IL-6, IL-18, TNF-α and CRP) detected in this study showed significantly higher in all the PCOS groups compared to the control group (*P* < 0.01, Fig. 4), and all these factors were significantly decreased in the lower supplementation of glutamine (*P* < 0.01).

### Serum oxidative stress factors

**Fig. 5:**
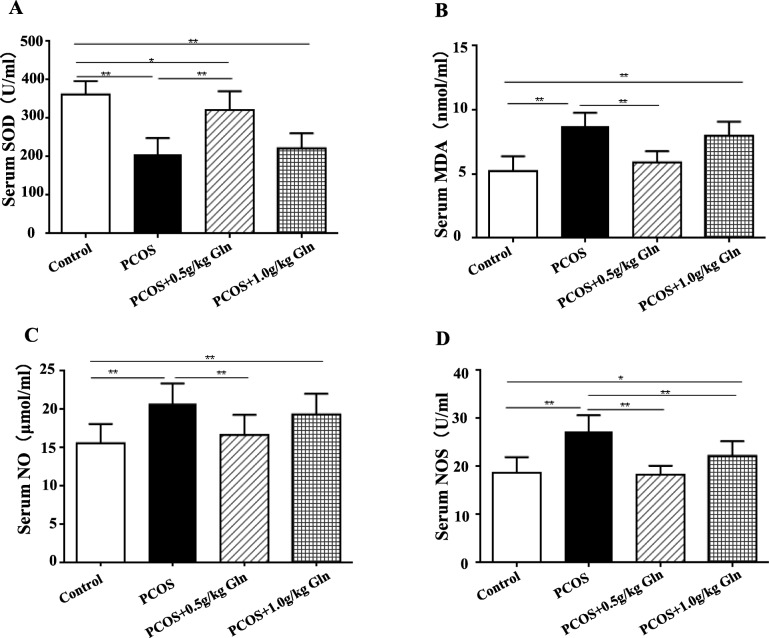
The serum concentrations of oxidative stress factors. **a**: Serum SOD levels; **b**: Serum MDA levels; **c**: Serum NO levels; **d**: Serum NOS levels. *n* = 10, *P < 0.05, **P < 0.001

Serum concentrations of SOD were significantly lower in PCOS groups (*P* < 0.01, Fig. 5a) compared to the control group, and increased significantly with the lower supplementation of glutamine (*P* < 0.01). Serum concentrations of MDA, NO and NOS were significantly higher in PCOS group (*P* < 0.01, Fig. 5b, c, d) compared with the control group, and significantly decreased to the comparative levels of control group with the lower supplementation of glutamine (*P* < 0.01).

## Discussion

PCOS is a common, clinically heterogeneous endocrine disorder, which is associated with endocrinopathy and metabolic abnormalities [10]. Ovulatory dysfunction, polycystic ovarian morphology, insulin resistance and hyperandrogenism are the typical characteristics [2]. More and more studies found that PCOS is associated with chronic inflammation and oxidative stress [2–4, 11]. Glutamine is the most abundant free amino acid in human circulation [12]. It is an important source of energy and a precursor of glutathione, and plays an essential role in several metabolic processes, such as protein and nucleic acid synthesis [13], and it also has anti-inflammatory and antioxidant effects as reported by previous studies [7, 8].

In the present study, the hormones and ovarian morphology changes, as well as insulin resistance in the DHEA-induced PCOS and PCOS+glutamine rats were in accordance with the characteristic of PCOS patients as previous studies [2, 9]. Significant decrease in the level of some amino acids including glutamine was observed in plasma in PCOS patients compared with the controls, these changes reflect the various metabolic pathways that participate in physiological functions [10]. However, there are rare studies about glutamine on hormones and ovaries. Olaniyi et al. [12] found that glutamine can attenuate the decrease of the serum concentrations of FSH, LH and T in cadmium-treated male rats for a consecutive 30 days’ administration. Gholipour et al. [14] reported that diet supplementing with L-glutamine for 40 days significantly increased levels of FSH and LH in guinea fowls. Our study also showed that T concentration was increased by the supplementation of glutamine in PCOS rats, and it increased with the dosage of the glutamine, but the LH concentration was decreased by supplementation of glutamine, even more, the concentration of FSH, E2, P and the ovaries didn’t show obvious changes among these different groups. Meanwhile, the insulin resistance situation was also not ameliorated in the PCOS groups. That may due to the short duration of glutamine administration (only twice in 1 day in this study), and it may need a longer time to recover the changes of hormones and ovarian morphology in PCOS.

Previous studies reported that there is low-grade inflammation associated with PCOS [10, 15], and our study found that serum inflammation factors levels, such as IL-6, IL-18, TNF-α and CRP, were significantly elevated in PCOS rats. Previous studies showed that the agents such as curcumin [15], resveratrol [16] which have anti-inflammatory effects can improve the low-grade inflammation state by decreasing the levels of TNF-α, IL-6 and CRP, including the PCOS rats. Glutamine also showed an anti-inflammation effect in inflammatory diseases, the treatment with glutamine has the potential to decrease of inflammatory cytokines [17].

Glutamine can attenuate muscle inflammation, maintaining the concentration of muscle TNF-α, IL-6 and IL-10 close to basal levels observed in the control group [8], glutamine supplementation following traumatic brain injury could inhibit NF-kB activation and down-regulate pro-inflammatory cytokine expression (TNF-α, IL-1b and IL-6) [18], and it also could decrease intestinal and plasma TNF-α in the induced intestinal inflammation rats [19]. Moreover, glutamine could decrease IL-6 in the intestine in human volunteers [20]. In the present study, the serum concentrations of IL-6, IL-18, TNF-α and CRP in PCOS rats were significantly decreased by supplementation of glutamine, especially in the 0.5 g/kg group, which indicated glutamine could also attenuate the low-grade inflammation of PCOS.

A lot of studies have revealed that oxidative markers in circulation are significantly increased in PCOS patients compared with the normal controls and a crucial role in the PCOS pathogenesis is played by oxidative stress [3, 4]. Oxidative stress is considered as the imbalance between oxidants and antioxidants and the increased production of reactive oxygen species (ROS) [4]. In the present study, some serum oxidative markers were detected and the results showed that serum concentrations of SOD were significantly lower in PCOS groups, MDA, NO and NOS were significantly higher in PCOS group compared with the control group, which was consistent with previous study on PCOS patients [21]. These findings dedicated that there is an oxidative stress in these DHEA-induced PCOS rats. Furthermore, results of the present study showed that the supplementation of 0.5 g/kg glutamine in PCOS rats could significantly increase the serum concentrations of SOD and maintain the concentrations of MDA, NO and NOS to basal levels observed in the control rats, all of these showed the antioxidant effects of glutamine. The effects of glutamine on improving antioxidant ability have been indicated in previous studies. Nemati et al. [7] reported that supplementation of glutamine can reduce oxidative stress and improve the antioxidant system of healthy adult males after an exhaustive exercise. Szpetnar et al. [22] found that glutamine is effective in reducing manganese-induced oxidative stress by increasing glutathione peroxidase and SOD activity and decreasing in MDA level.

## Conclusions

The present study demonstrates that DHEA-induced PCOS rats showed the similar hormones and ovarian morphology changes as PCOS patients, and there is low-grade inflammation and oxidative stress in PCOS rats. The supplementation of 0.5 g/kg glutamine could effectively ameliorate the inflammatory and oxidative stress conditions of PCOS, however, it may need a longer administration time of glutamine to recover the changes of hormones and ovarian morphology in PCOS.

## Data Availability

The datasets used and analysed during the current study are available from the corresponding author on reasonable request.

## Abbreviations

PCOS: Polycystic ovary syndrome
DHEA: Dehydro-epiandrosterone
T: Testosterone
LH: Luteinizing hormone
E2: Estradiol
FSH: Follicle stimulating hormone
P: Progesterone
CRP: C-reactive protein
IL-6: Interleukin 6
IL-18: Interleukin 18
TNF- α: Tumor necrosis factor α
SOD: Superoxide dismutase
MDA: Malondialdehyde
NO: Nitric oxide
NOS: Nitric oxide synthase
